# Retropulsion force in laser lithotripsy—an in vitro study comparing a Holmium device to a novel pulsed solid-state Thulium laser

**DOI:** 10.1007/s00345-021-03668-8

**Published:** 2021-03-23

**Authors:** Ralf Petzold, Arkadiusz Miernik, Rodrigo Suarez-Ibarrola

**Affiliations:** Department of Urology, Faculty of Medicine, University of Freiburg-Medical Centre, Hugstetter Str. 55, 79106 Freiburg, Germany

**Keywords:** Laser, Lithotripsy, Holmium, Thulium, Ho:YAG, Tm:YAG, Stone disease, Retropulsion, Repulsion, Propulsion

## Abstract

**Purpose:**

To investigate retropulsion forces generated by two laser lithotripsy devices, a standard Ho:YAG and a new pulsed solid-state Thulium laser device.

**Materials and methods:**

Two different Dornier laser devices were assessed: a Medilas H Solvo 35 and a pulsed solid-state Thulium laser evaluation model (Dornier MedTech Laser GmbH, Wessling, Germany). We used a 37 °C water bath; temperature was monitored with a thermocouple/data-logger. Representative sets of settings were examined for both devices, including short and long pulse lengths where applicable. For each setting, ten force values were recorded by a low-force precision piezo sensor whereby the laser fibre was either brought into contact with the sensor or placed at a 3 mm distance.

**Results:**

The mean retropulsion forces resulting from the new Tm:YAG device were significantly lower than those of the Ho:YAG device under all pulse energy and frequency settings, ranging between 0.92 and 19.60 N for Thulium and 8.09–39.67 N for Holmium. The contact setups yielded lower forces than the distance setups. The forces increased with increasing pulse energy settings while shorter pulse lengths led to 12–44% higher retropulsive force in the 2.0 J/5 Hz comparisons.

**Conclusion:**

The Tm:YAG device not only significantly generated lower retropulsion forces in all comparisons to Holmium at corresponding settings but also offers adjustment options to achieve lower energy pulses and longer pulse durations to produce even lower retropulsion. These advantages are a promising add-on to laser lithotripsy procedures and may be highly relevant for improving laser lithotripsy performance.

## Introduction

Over the past two decades, Holmium:Yttrium–Aluminium–Garnet (Ho:YAG) laser lithotripsy has become the gold-standard for treating urinary stone disease due to its safe, efficient and versatile properties. However, despite its advantages, Ho:YAG lithotripsy has significant inherent limitations such as stone retropulsion which results in a reduction of the stone ablation rate, increased operative time, decreased stone-free rates, and need for ancillary procedures with concomitant morbidity and healthcare costs [1]. It has been reported that 3–15% of distal ureteral stones and 28–60% of proximal stones undergo retropulsion [2], therefore, minimizing retropulsion is highly relevant to improve laser lithotripsy performance and to avoid undesired outcomes.

The laser-generated retropulsion force inducing stone migration is caused by stone particles being released from the lasers’ impact crater [3], and by fluid turbulences during gas bubble formation at the laser fibre’s tip when there is no impact on the stone. The emitted laser light itself generates almost no force since the radiation pressure is negligible. Retropulsion is, therefore, mainly generated by the pressure wave created by the gas bubble and released stone fragments. Considerable scientific data has been published on the optimal settings for low retropulsion revealing that applying thin fibres, long pulse durations, and low pulse energies leads to low retropulsion [1, 4].

However, in most in vitro studies, retropulsion is investigated via indirect methods which include pendulums [5, 6], angled glass channels and flat glass setups [1, 3, 4, 7–21], and vertical set-ups [22, 23]. Through these indirect methods, there are several factors potentially influencing the results such as fluid dynamics, the impact’s exact location, and the test body’s irregular surface and composition. Hence, the comparison of results between the studies proves to be difficult due to varied experimental setups and test materials.

In this study, we used a low-force and high-precision piezo sensor to objectively measure retropulsion forces of a Ho:YAG laser and an evaluation model of a novel solid-state Thulium laser. To compare both laser devices in in-vitro experiments, we focused on laser-generated retropulsion forces to exclude potential sources of deviation and to ensure reproducible results.

## Materials and methods

The laser devices examined and compared were a Medilas H Solvo 35 Ho:YAG laser and the evaluation model of a diode-pumped pulsed solid-state Thulium laser, which should not be confused with a Thulium fibre laser (TFL). Both devices are manufactured by Dornier (Dornier MedTech Laser GmbH, Wessling, Germany). The Thulium device provides powers of up to 120 W, while it is also operable on a standard 230 V single-phase power supply. Its possible single pulse energies range is 100–3000 mJ with frequencies up to 200 Hz, which promise both effective low retropulsion dusting and precise fragmentation.

A 15 l glass water-tank was used along with a heating rod (thermocontrol 3604, Eheim, Deizisau, Germany) which kept at a constant 37 °C temperature. Temperature was monitored by a type K thermocouple with a real-time data-logger TC-08 (PICO Technologies, Cambridgeshire, United Kingdom) and evaluated by MatLab (The Mathworks Inc., Natick, Massachusetts, USA). 400 µm laser fibres were used and freshly cut before each experimental run (Dornier MedTech GmbH, Wessling, Germany). Fibres which were used up and thus too short for our setup were replaced. The 400 µm fibre size was chosen to allow for exceeding powers of the Thulium device, as 272 µm fibres are only certified for 30 W. The laser energy was checked with a StarBright power/energy meter (Ophir, Jerusalem, Israel), and fibers that deviated more than ± 50 mJ from the set value were replaced/recut.

**Fig. 1:**
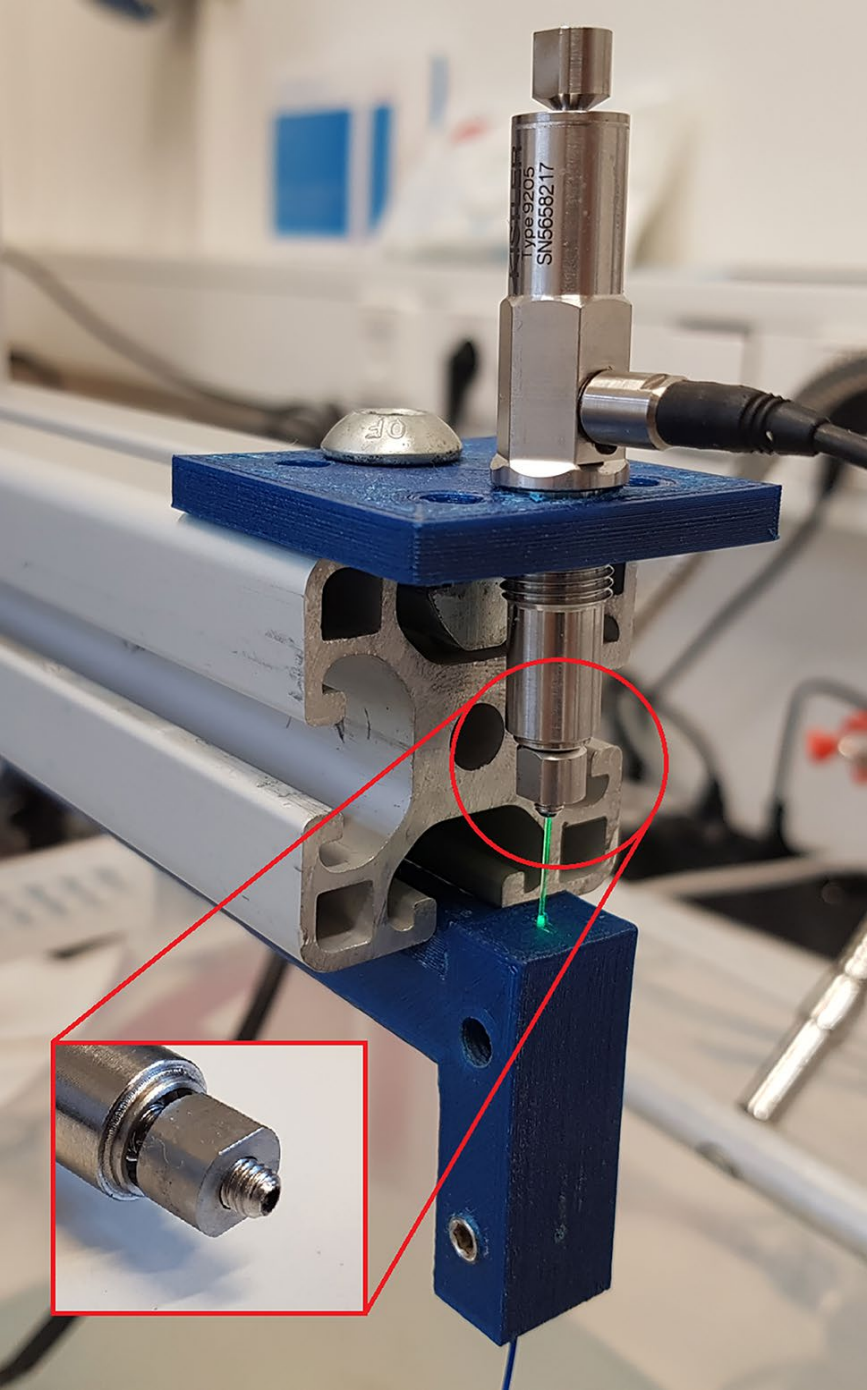
Experimental setup with piezo sensor and laser fibre

To measure the exerted force, we used a low-force precision piezo sensor type 9205 and a data acquisition unit 5165A which has a 200 kHz measuring frequency and a sensor resonance frequency of over 10 kHz, both fabricated by Kistler (Kistler Instrumente GmbH, Sindelfingen, Germany). Sensor and laser fibre were held inside the water-bath by an aluminium framework and 3d-printed mountings (Fig. 1). Since both laser devices are capable of ablating metal, the piezo sensor was protected from direct impacts by placing the laser fibre against a grub screw that was mounted on the sensor. There were two types of experimental setups. In the first, the fibre was placed in direct contact with the metal screw/sensor, while in the second setup the fibre was placed 3 mm away from the sensor. The experiments were repeated ten times for each setup and each laser setting.

The examined laser settings cover the range of possible Thulium and Holmium power settings for comparison (Table 1). We attempted to adjust pulse durations, but this is only technically feasible to a certain degree since the Thulium device’s pulse durations tend to be longer than the Ho:YAG device’s.

**Table 1:** Summary of measurements in contact and distance setups, direct comparisons in the upper part, additional investigations of Thulium in the lower part

Device	Settings				Contact				Distance			
	*f* [Hz]	*t* [µs]	*E* [J]	*P* [W]	#	E_m [J]	F [N]	SD_F [N]	#	E_m [J]	F [N]	SD_F [N]
Holmium	5	270	1,0	5	1	1,002	8,09	0,49	12	1,000	11,91	0,97
Thulium	5	412	1,0	5	2	1,000	**7,53**	0,25	13	0,998	**10,96**	0,42
Holmium	5	290	2,0	10	3	2,010	16,65	0,56	14	2,011	32,60	1,61
	5	370	2,0	10	4	2,007	11,60	0,64	15	2,009	28,18	1,68
Thulium	5	648	2,0	10	5	2,001	10,33	0,71	16	2,001	16,42	0,62
	5	1020	2,0	10	6	2,002	**7,70**	0,65	17	2,005	**14,64**	0,99
Holmium	10	440	3,0	30	7	3,021	32,31	2,76	18	3,019	39,67	1,62
Thulium	10	888	3,0	30	8	2,998	**17,93**	1,93	19	2,999	**19,60**	0,88
Thulium	25	269	0,1	2,5	9	0,100	0,92	0,14	20	0,100	1,76	0,16
	10	964	2,0	20	10	2,001	9,72	0,54	21	2,000	16,83	1,30
	20	1000	2,0	40	11	2,001	7,90	0,81	22	2,002	14,74	0,76

Bold lowest values in direct comparisons

*f* frequency, *t* optical pulse duration, *E* single pulse energy, *P* power, *E_M* measured single pulse energy, *F* measured retropulsion force, *SD_F* standard deviation of the measured force

Statistical calculations were performed using Microsoft Excel (Microsoft Corporation, Redmond, USA). Student’s *t* test was used in MatLab to evaluate the differences between continuous variables from different sets of experiments. Statistically significant differences were considered for *p* < 0.05.

## Results

Altogether we obtained 220 maximum force values and 88 energy meter readings in 22 sets of experiments in both the contact and 3 mm distance setups. Table 1 summarizes the retropulsion forces obtained when comparing both laser devices in the contact and non-contact experiments. All stated comparisons were significant if not stated otherwise.

In the contact setup, the Thulium device generated the lowest retropulsion force value of 0.92 N at 0.1 J/25 Hz/269 µs (Experiment #9), and the highest value of 17.9 N at 3.0 J/10 Hz/888 µs (#8). Conversely, Holmium’s retropulsion forces ranged between 8.1 N at 1.0 J/5 Hz/270 µs (#1) and 32.3 N at 3.0 J/10 Hz/440 µs (#7). In direct comparisons at 1.0 J/5 Hz, Holmium and Thulium produced comparable values of 8.1 N and 7.5 N, respectively, with a slight but significant advantage for Thulium (*p* < 0.05). At 2.0 J/5 Hz and 3.0 J/10 Hz, the Holmium laser delivered significantly greater retropulsion forces (+ 74.6%) than the Thulium device: 11.6 N vs 7.70 N and 32.3 N vs 17.93 N, respectively (*p* < 0.05 each). Regarding the influence of shorter pulse durations on retropulsion forces at 2.0 J/5 Hz, a 43.5% increase was obtained for Holmium when comparing 290–370 µs (*p* < 0.05), and a 34.2% increase for Thulium in 648 µs vs 1020 µs comparisons (*p* < 0.05).

The distance setup generated higher forces than did our contact experiments ranging from 1.76 N for Thulium at 0.1 J/25 Hz (#20) to 39.67 N for Holmium at 3.0 J/10 Hz (#18). When the distance and contact experiments were compared by percentage increase in retropulsion forces, values of 9.3% (#8 vs #19 Tm 3.0 J/10 Hz, *p* < 0.05) to as high as + 143% (#4 vs #15 Ho 2.0 J/5 Hz/370 µs, *p* < 0.05) were obtained, with a + 72% mean over all experiments. Otherwise, the considerations about higher retropulsion in short pulse durations in the distance setup are analogous to the contact setup, which can be observed in various 2.0 J settings. For Holmium, the 2.0 J/5 Hz/290 µs setting yielded + 15.7% higher forces than a pulse duration of 370 µs, while for Thulium a + 12.2% increase was found for 648 µs vs 1020 µs at 2.0 J/5 Hz (*p* < 0.05 for each comparison).

In general, higher pulse energies correspondingly generate higher retropulsion forces. Shorter pulse durations in conjunction with fixed energy/frequency also lead to more retropulsion—evident when comparing the results of these settings for each technology separately (#3–6, #14–17). Concerning frequency dependency, decreasing retropulsion forces can be found for higher frequencies of 20 Hz at 2.0 J, see #6/11 and #17/22 at approximately the same pulse duration, however, these results did not reach a level of significance.

## Discussion

To the best of our knowledge, this is the first time a high precision piezo sensor is used to measure the retropulsive forces created by different laser lithotripsy devices. Other research groups have evaluated retropulsion by measuring stone displacement, velocity or a combination of both [4, 5, 7, 10, 23–25], not allowing for direct comparisons to our study. We like to promote the idea of using such high precision force sensors to evaluate retropulsive forces. The sensor’s capabilities concerning temporal resolution (≥ 25 kSps) and precision are perfectly suited to measure retropulsion of laser pulses. Attention has to be payed to the use of sacrificial screws or plates on the sensor’s tip, as lithotripsy lasers can easily ablate metal due to the enormous temperatures on the laser fibre tip, as shown for thulium laser lithotripsy devices by Wilson et al. [26].

The resulting forces are not only dependent on laser settings, they also reveal considerable variance in a series of pulses. This phenomenon depends on several factors, i.e., the water temperature between laser fibre tip and sensor, bubble formation, slight distance variations from laser fibre to sensor, the water turbulence between sensor and laser fibre in the distance setup, and variations in the laser pulses themselves. All these aspects are influenced by continuous laser activation and result in force variations. However, standard deviations are consistently small with a maximum of ± 15.4% of the mean value which was observed in the minimal 0.1 J Thulium setting.

The fact that shorter pulse durations produce higher retropulsive forces [1, 4] must apply across different devices since a faster energy release signifies higher energy density, thus causing larger and shorter-lasting vapour bubbles. For instance, similar force values were obtained with the 1.0 J/5 Hz setting at 270 µs for Holmium compared to 412 µs for Thulium (Ho vs Tm: contact 8.09 N vs 7.53 N, distance 11.91 N vs 10.96 N). We can thus infer that with the same pulse duration, the Thulium device would likely generate greater retropulsion forces attributable to its higher absorption coefficient in water. At 37 °C water temperature, the Thulium laser’s absorption coefficients were calculated to 5888 m^−1^ at 2013 nm wavelength, and 3198 m^−1^ for the Holmium device at 2080 nm wavelength. This results in an approximately 84% longer absorption time for Holmium compared to Thulium. The energy density at the fibre tip, therefore, depends on both the pulse duration in which the energy is emitted and on the absorption coefficient. Both influences the shape and size of the gas bubble and thus the resulting pressure and retropulsion.

In this investigation, we opted not to address the force generated by the influence of released stone particles first reported by Choi et al. [3] since we measured the force directly at the sensor tip and without stone models. Retropulsion also occurs when a stone is not hit directly, but is moved by the pressure generated by the gas bubble and turbulences. This applies especially to the popcorn technique, where these forces play an important role in positioning the stone in front of the laser fibre [27].

Li et al. presented a setup to directly measure retropulsive forces of a VersaPulse^®^ PowerSuite^™^ (Lumenis Ltd., Yokneam, Israel). Their setup consisted of a Mark-10 force gauge which was inserted through the wall of a water-bath and a glass tube in which a stone model was put in direct contact with the fibre. Although the authors reported maximum forces of up to 0.907 N for 0.6 J/5 Hz, we could not corroborate their findings [28]. In our study, these forces were measured in the Thulium laser’s 0.1 J single pulse energies. With the Holmium laser at 1.0 J, we obtained values of 7.5 N in the contact setup and 10.9 N in 3 mm distance, which is 6–9 times higher than the reported findings at 0.6 J. These differences may be attributed to the measuring device. The Mark-10 force gauge works with strain gauge sensors. First, the force gauge’s signal elevations are too slow and these sensors are unable to react sufficiently quickly to the short laser pulses (statement by two Mark-10 and Kistler employees). Second, the sensor in the presented setup appears to have been glued onto the experimental setup and, therefore, cannot be pushed into the device to adequately measure the applied force.

Retropulsion is an important factor influencing fragmentation efficiency. Thus, high single pulse energies at low frequencies and short pulse durations have often been proposed and used for fragmentation [29], but this can lead to high retropulsion as the stone is increasingly ablated. Newer Holmium laser devices such as the Lumenis Moses^™^ Pulse 120H currently deliver frequencies of 80 Hz, while the new Thulium laser evaluation model delivers a maximum of 200 Hz, falling between the Ho:YAG and the TFL technologies. With possible pulse energies of 100 mJ and 200 Hz frequency, however, fast, low-retropulsion dusting and fragmentation may be conceivable.

Comparing our contact and distance setups, the latter tended to reveal higher retropulsion forces. Lithotripsy should therefore always be performed by guiding and activating the laser in contact with the stone to reduce retropulsion. In our experience, direct stone contact increases fiber degradation, but dusting efficiency does not diminish significantly with fibre wear. Furthermore, in a contact setup, more energy is presumably transmitted directly to the stone and less energy is transferred into the surrounding water bath with a reduced effect on the targeted stone.

The diameter of the laser fibre used often depends on the type of approach, for example, larger-sized fibres for percutaneous nephrolithotomy and smaller for retrograde intrarenal surgery. We expect the general physical principle of increased retropulsion in rising pulse energy and decreasing pulse duration to always apply. At the moment it is unclear to what extent the absolute values found with 400 µm fibres can be extrapolated to fibres of different sizes. Differing energy density/distribution on the laser fibre tip in combination with the resulting gas bubble shapes will directly affect retropulsion. Further studies are needed that compare Ho:YAG and Tm:YAG with laser fibres of varying diameter to determine the impact of fibre calibre on retropulsion, as well as on lithotripsy efficiency and fragment size.

A particular strength of the study is that we determined real-life retropulsion force values, thereby avoiding less accurate measurements taken with a pendulum or by displacing stone models. For this reason, we recommend future studies to be performed in a similar fashion to allow inter-study comparisons of laser devices and fibres. Limitations to this study were that we did not use stone models and thus could not measure the proportional force potentially generated by the displacement of stone particles. Stone removal would have to be compensated by steadily advancing the fibre to obtain a constant distance between fibre and stone, thus generating reproducible force measurement values. To validate our findings, retropulsive forces should also be measured when using real or artificial stone samples as a more realistic model. Another limitation is the exclusive use of 400 µm fibres, which only allows reasonable assumptions regarding 200/272 µm fibers. Similar results seem highly likely but need to be verified in further studies. Regarding new vs. freshly cut fibres, we did not experience any deviation in our results. Furthermore, the Holmium device precluded a specific adjustment of the pulse duration; therefore, a 1:1 comparison of any two settings was not possible. This is due to the Holmium’s shorter pulse duration compared to the Thulium laser. These considerations should be acknowledged in further investigations.

## Conclusion

At the same energy and pulse frequency settings, the new Thulium device produces significantly lower retropulsion forces than the current Ho:YAG technology, likely being related to the Thulium laser’s longer pulse lengths. The Thulium device offers a long pulse setting that is more advantageous for considerably reducing retropulsion forces by 7–55% in the energy and frequency settings tested. Moreover, the Thulium laser provides additional adjustment options of the pulse duration that enable even lower retropulsion. The potential to combine frequencies up to 200 Hz with low single pulse energies e.g. 100 mJ promises highly efficient dusting with minimal retropulsion. The advantages of this new technology are a promising addition to laser lithotripsy procedures, especially when treating stones in challenging locations, such as the ureteropelvic junction and in close proximity to the lower calyx, where low retropulsion and effective disintegration are desired to minimize operative time and health care costs. Finally, since significantly less retropulsion was detected with the fibre in contact with the sensor, we recommend lithotripsy to be performed under these conditions.
